# Zoonotic transmission of *Teladorsagia circumcincta* and *Trichostrongylus species* in Guilan province, northern Iran: molecular and morphological characterizations

**DOI:** 10.1186/s12879-020-4762-0

**Published:** 2020-01-10

**Authors:** Keyhan Ashrafi, Meysam Sharifdini, Zahra Heidari, Behnaz Rahmati, Eshrat Beigom Kia

**Affiliations:** 1 Department of Medical Parasitology and Mycology, School of Medicine, Guilan University of Medical Sciences, Rasht, Iran; 2 Department of Medical Microbiology and Parasitology, School of Medicine, Ardabil University of Medical Sciences, Ardabil, Iran; 3 Department of Medical Parasitology and Mycology, School of Public Health, Tehran University of Medical Sciences, Tehran, Iran

**Keywords:** *Trichostrongylus colubriformis*, *Trichostrongylus vitrinus*, *Teladorsagia circumcincta*, Human, Iran

## Abstract

**Background:**

Parasitic trichostrongyloid nematodes have a worldwide distribution in ruminants and frequently have been reported from humans in Middle and Far East, particularly in rural communities with poor personal hygiene and close cohabitation with herbivorous animals. Different species of the genus *Trichostrongylus* are the most common trichostrongyloids in humans in endemic areas. Also, *Ostertagia* species are gastrointestinal nematodes that mainly infect cattle, sheep and goats and in rare occasion humans. The aim of the present study was to identify the trichostrongyloid nematodes obtained from a familial infection in Guilan province, northern Iran, using morphological and molecular criteria.

**Methods:**

After anthelmintic treatment, all fecal materials of the patients were collected up to 48 h and male adult worms were isolated. Morphological identification of the adult worms was performed using valid nematode keys. Genomic DNA was extracted from one male worm of each species. PCR amplification of ITS2-rDNA region was carried out, and products were sequenced. Phylogenetic analysis of the nucleotide sequence data was performed using MEGA 6.0 software.

**Results:**

Adult worms expelled from the patients were identified as *T. colubriformis*, *T. vitrinus* and *Teladorsagia circumcincta* based on morphological characteristics of the males. Phylogenetic analysis illustrated that each species obtained in current study was placed together with reference sequences submitted to GenBank database.

**Conclusions:**

The finding of current study confirms the zoonotic aspect of *Trichostrongylus* species and *T. circumcincta* in inhabitants of Guilan province. The occurrence of natural human infection by *T. circumcincta* is reported for the first time in Iran and the second time in the world.

## Background

Trichostrongyloid nematodes (Nematoda: Trichostrongyloidae) are significant parasites of the digestive tract of herbivorous animals. Various genera of these nematodes are prevalent in ruminants in different countries throughout the world [1]. They have an important veterinary role due to their impact on animal health and severe economic losses in herbivores [1]. Among the Trichostrongyloidae, *Trichostrongylus* is the most common genus infecting humans in endemic regions [1]. Other genera of trichostrongyloid nematodes such as *Haemonchus contortus, Marshallagia marshalli, Nematodirus abnormalis, Ostertagia ostertagi* and *O. circumcincta* (*Teladorsagia circumcincta*) have been reported in humans, particularly in Iran and Azerbaidjan, of former Soviet Union [2–5].

*Trichostrongylus* species are primarily parasites of livestock with a worldwide distribution but they have also been reported from humans in Middle and Far East and Africa, particularly in rural communities with poor personal hygiene and close cohabitation with herbivorous animals [2, 4, 6–8]. Epidemiological studies have demonstrated trichostrongylosis as a prevalent parasitic infection in domestic ruminants and humans in different regions of Iran [2, 9]. Prevalences of 53.6, 46.8, 42.4, 38.7, 37 and 35.8% have been reported in ruminants from Isfahan, Khuzestan, Mazandaran, Kermanshah, Hormozgan and West Azerbaijan provinces and 71, 67, 42, 37, 19, 8, 2 and 1% in humans in Khuzestan, Isfahan, Tehran, Hormozgan, Kermanshah, Mazandaran, West Azerbaijan and Sistan & Baluchestan provinces in the past decades respectively [2]. Recent studies indicate a decreasing trend in the prevalences of trichostrongylosis in both animals and humans in Iran. However, human trichostrongylosis is still a health problem in some regions of the country. The prevalences of 18.1% [9], 3.05% [6] and 2.1% [10] have been reported from Khouzestan, Guilan, and Mazandaran provinces in recent years respectively.

Until now, ten species of the genus *Trichostrongylus* have been reported from humans in various parts of Iran, which is unique in the world when the number of collected species is taken into consideration. Among these species, *T. colubriformis* and *T. orientalis* were more common in past decades in the country [2, 6, 11–13], but recent studies in the north of Iran have shown *T. colubriformis* as the predominant species in infected individuals [6, 7, 14, 15]. Humans mostly acquire the infection through consumption of fresh vegetables contaminated with infective filariform larvae [6, 7]. Trichostrongylosis is usually asymptomatic in low intensity infections, but abdominal pain, diarrhea, anorexia, nausea, weakness, mild anemia, low-grade peripheral eosinophilia, pulmonary and cutaneous symptoms are the most common manifestations in heavy infections [8, 16, 17].

*Ostertagia* species, commonly known as the brown stomach worm, are gastrointestinal nematodes that infect cattle, sheep and goats and other wild ruminants worldwide, especially in temperate and cool climates. *O. ostertagi* mainly infects cattle, but also sheep, goats and other domestic and wild ruminants [1]. It has been reported from two persons in central part of Iran and one person in Azerbaijan of former Soviet Union [3]. *T. circumcincta,* which was originally placed in the genus *Ostertagia,* is a parasitic nematode of gastric glands of the stomach that infects sheep and goats all around the world and leads to weight loss, decreased wool production and death [18]. The prevalence rates of 17.3 to 47.2% for *T. circumcincta* have been reported in sheep and goats in different provinces of Iran [19–21]. A single human case of *T. circumcincta* has been reported from Azerbaijan of former Soviet Union in the past decades [5].

Detection of characteristic eggs in stool samples of humans and animals is a routine diagnostic method for trichostrongyloid infections [22]. However, classification of *Trichostrongylus* species and other genera of trichostrongyloid nematodes are based on differences in the morphology of male worms [19].

Recently, PCR-based tools are applied for reliable identification and phylogenetic analysis of nematodes. Based on the recent studies, the internal transcribed spacer 2 (ITS2) region of ribosomal DNA is a useful tool for analyzing genetic variations and phylogenetic relationships in nematodes belonging to the Trichostrongylidae family [6, 7, 20, 21]. The aim of the present study was identification, molecular characterization and phylogenetic analysis of human specimens of *Trichostrongylus* and *Teladorsagia* obtained from the patients during a familial infection in Langroud district of Guilan province based on ITS2 region of ribosomal DNA.

## Methods

### Study area

**Fig. 1:**
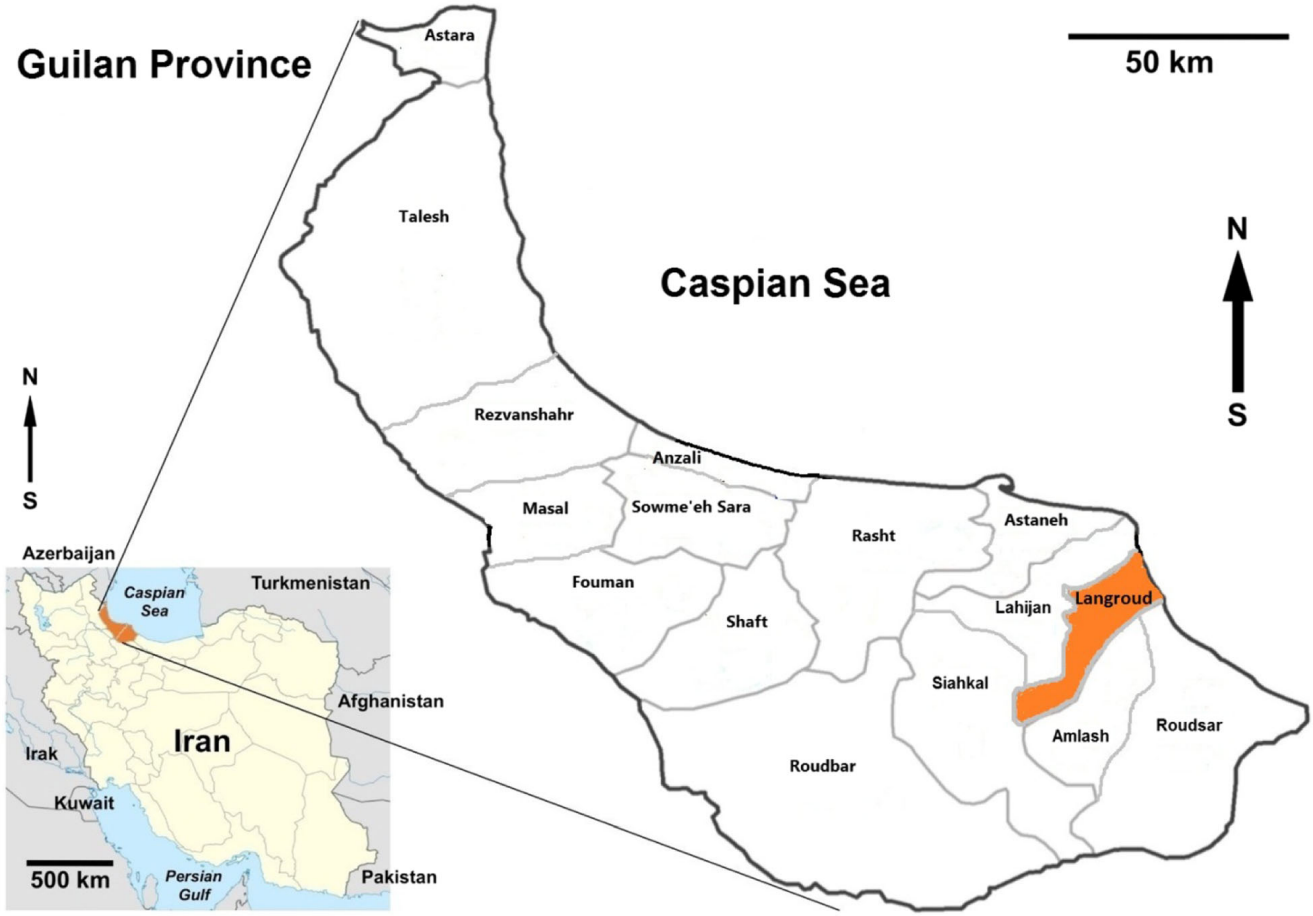
Map of Iran showing geographical location of Guilan province and the study area, Langroud district (https://ars.els-cdn.com/content/image/1-s2.0-S2352340918313313 gr1.jpg)

Guilan province is one of the 31 provinces of Iran located at the southern littoral of Caspian Sea in northern Iran and is comprised of sixteen districts. Langroud district with an area of 480 km^2^ (37° 11′ 49″ N, 50° 9′ 13″ E) is located at the eastern part of the province (Fig. 1). Overall, 140,686 (70,675 males and 70,011females) were recorded to live in 49,351 families in the study region in 2016.

### Sample collection and morphological identification

Seven members of a family living in Langroud district, located at the eastern part of Guilan province, were diagnosed to have trichostrongylosis following comprehensive clinical and laboratory examinations. Abdominal, epigastric and right upper quadrant pain, diarrhea, low grade fever, rigors especially at nights, allergic manifestations including urticaria associated with itching, flatulence and weight loss were the most important clinical manifestations declared by the patients. Hypereosinophilia ranging from 25 to 60% were seen. No parasite ova were obtained following examination of three stool samples on every other day using formalin-ether and Kato-Katz techniques. Since the patients were living in a high-risk area of human fasciolosis they were treated for acute disease using Egaten (10 mg/kg). No evidence of treatment success was observed about four weeks after drug therapy. Surprisingly, all patients shed *Trichostrongylus* ova following a new set of three stool examination. The infected individuals were treated with a single dose of albendazole (400 mg) followed by 200 mg of mebendazole per day for 3 days [14]. All fecal materials of the patients was collected up to 48 h following chemotherapy in large screw capped plastic containers in 70% alcohol. Fecal materials were homogenated slowly and filtered using tap water over a sieve of 100 mesh (150 μm) for collecting the adult worms. The isolated worms were put in 70% alcohol for morphological and molecular studies. Morphological identification of the adult worms was performed by putting the males in a mixture of lactophenol and azocarmine on a glass slide, using valid nematodes systematic keys [23]. The specimens were also photographed using a light microscope equipped with a digital camera (TUCSEN, TrueChrome II, China).

### DNA isolation and PCR amplification

A single male worm of each species isolated from the patients was processed for DNA extraction. The worms were washed with distilled water to remove ethanol and genomic DNA was extracted using Bioneer DNA extraction kit (Bioneer Corporation, Daejeon, Korea) according to the manufacturer’s instructions and stored at − 20 °C until PCR amplification.

PCR reactions were performed in 30 μL volumes containing 2 × red PCR premix (Ampliqon, Odense, Denmark), 20 pmol of each primer and 3 μL of extracted DNA. Primers NC1 (5-ACGTCTGGTTCAGGGTTGTT-3) and NC2 (5- TTAGTTTCTTTTCCTCCGCT-3) amplify a 328 base pair (bp) target of internal transcribed spacer 2 (ITS2) gene [24]. The thermal PCR profiles included an initial denaturation step at 95 °C for 6 min, followed by 35 cycles of denaturation at 94 °C for 45 s, annealing at 60 °C for 90 s, extension at 72 °C for 60 s, followed by a final extension at 72 °C for 5 min. Samples containing water instead of templates were used as negative control. DNA extracted from *T. colubriformis* isolate of sheep was used as positive control in PCR reaction. The PCR products were electrophoresed on a 1.5% of agarose gel and visualized using ethidium bromide on an UV Transilluminator and digitally photographed.

### Sequencing and phylogenetic analysis

PCR products were purified using a commercial purification kit (Bioneer, South Korea), according to the manufacturer’s instructions. Purified products were sequenced in both directions using identical forward and reverse primers as in the PCR. Sequence results were edited and analyzed by BioEdit software (http://www.mbio.ncsu.edu/bioedit/bioedit.html), and consensus sequences were compared with GenBank reference sequences using the BLAST system (http://www.ncbi.nlm.nih.gov/). The sequences of *T. colubriformis*, *T*. *vitrinus* and *T. circumcincta* were deposited in GenBank database under the accession numbers KF826913, KF872228 and KF989498, respectively.

Phylogenetic analysis was carried out using sequences obtained in this study along with relevant sequences deposited in the GenBank. A maximum likelihood tree was constructed based on the Tamura-Nei model and pairwise comparisons were made of the level of sequence differences within and among species using the MEGA 6.0 software. Bootstrap analysis was performed to determine the robustness of the finding based on 1000 replications.

## Results

### Parasitological findings

**Fig. 2:**
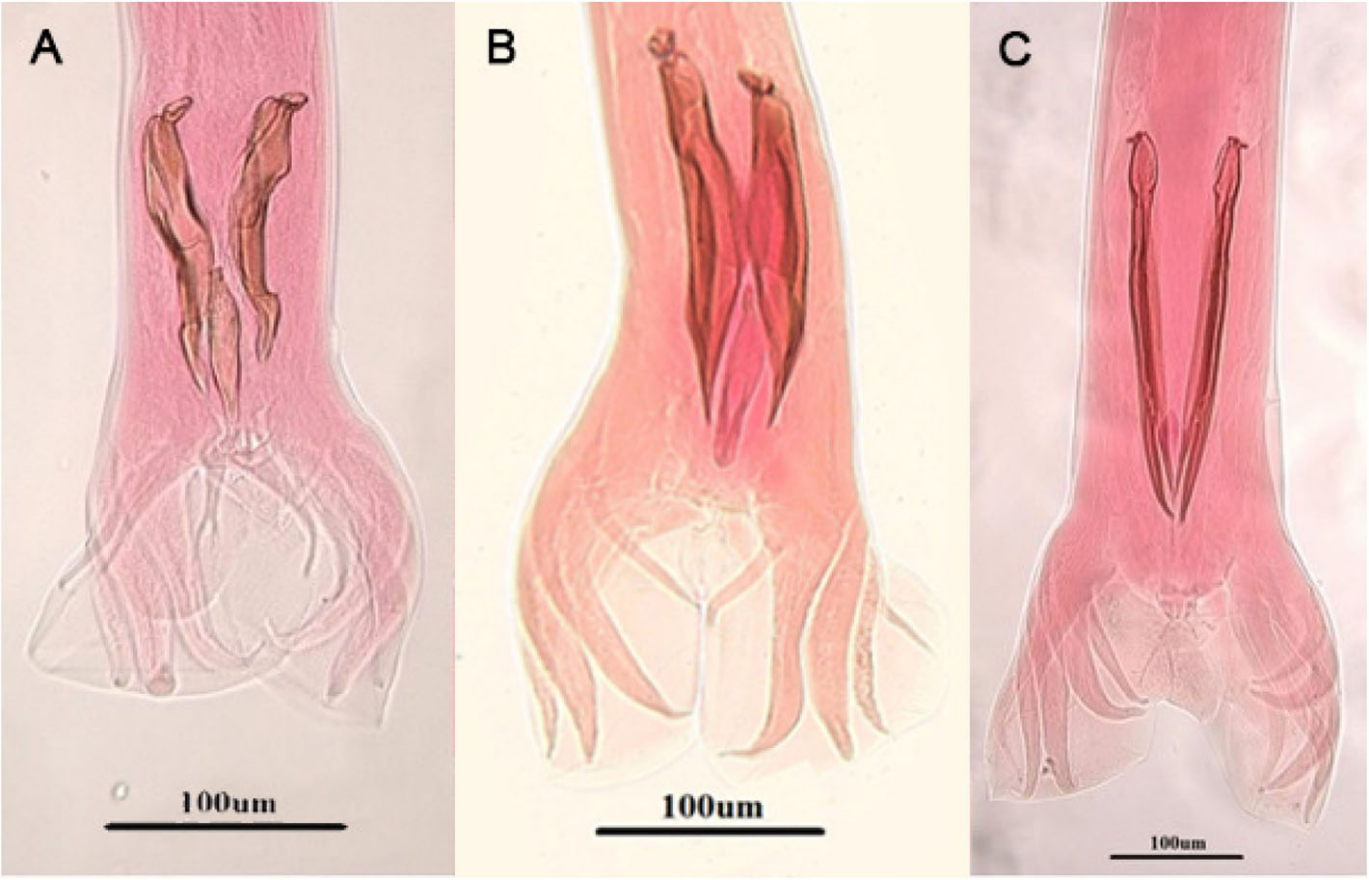
Light microscope view of trichostrongylid nematodes isolated from humans in Langroud district of Guilan province, northern Iran. Copulatory bursa and spicules of *Trichostrongylus colubriformis* **a**, *T. vitrinus* **b** and *Teladorsagia circumcincta* **c** (Scale bar: 100 μm)

Adult worms isolated from fecal materials of the patients were identified as *T. colubriformis*, *T. vitrinus* and *T. circumcincta* according to the morphology of bursa copulatrix and spicules of the males. The morphological characteristics of spicules and gubernaculum of *T. colubriformis*, *T. vitrinus* and *T. circumcincta* are illustrated in Fig. 2. Based on systematic references [23], a brief description of spicules of male worms which have been collected from the patients is presented here.

- ***T. colubriformis*:** Spicules are slightly unequal in lengths and have boat-shaped structure with a thick outgrowth capping at proximal end. Also the gubernaculum has a wavy curve with two bends when seen laterally.
- ***T. vitrinus:*** The spicules are small, near equal and straight with sharply tapering at distal ends.
- ***T. circumcincta*:** The spicules are equal and each one fitted two fins at the sides. The posterior end of the spicule is split into two branches of equal length.

Table 1 shows the information of the patients such as number and species of the male worms collected by parasitological method in each treated patient.

**Table 1:** Number and species of the male worms collected from seven infected individuals in Langroud district, Guilan province, northern Iran. Ova* = presence of ova before treatment; Ova** = presence of ova after treatment

Patients	ova*	ova**	Number of male worms collected following treatment			
			*Trichostrongylus colubriformis*	*Trichostrongylus vitrinus*	*Teladorsagia circumcincta*	Total
1	+	–	14	3	–	17
2	+	–	19	5	2	26
3	+	–	–	–	–	–
4	+	–	8	–	–	8
5	+	–	11	5	2	18
6	+	–	–	–	–	–
7	+	–	7	4	–	11

### Molecular findings

**Fig. 3:**
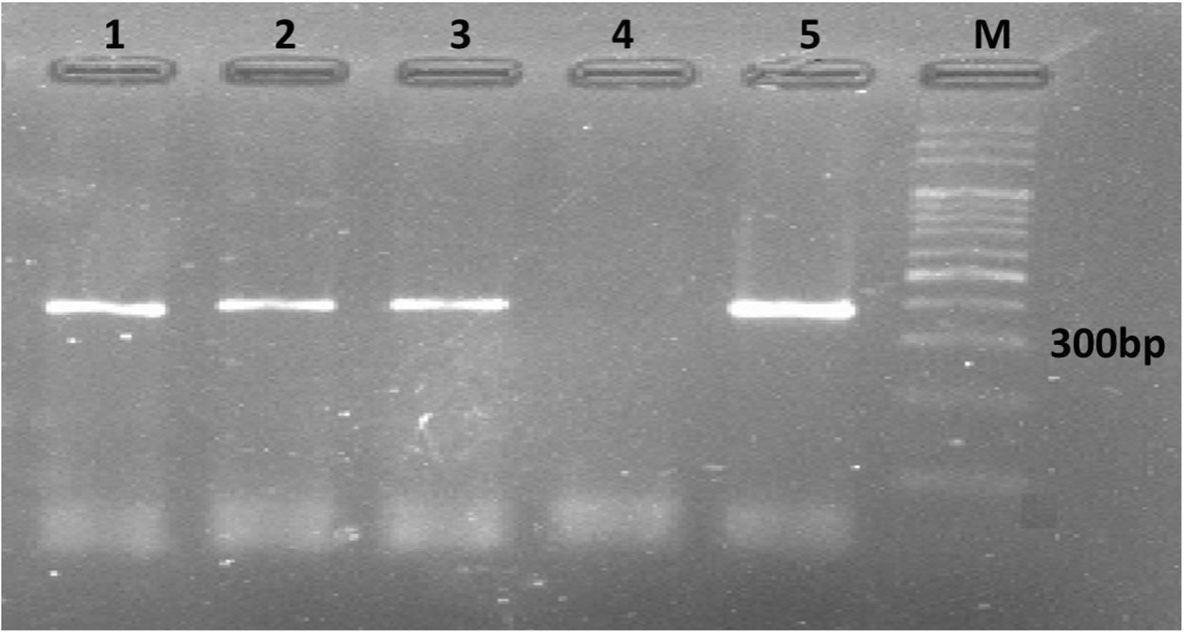
Agarose-gel electrophoresis of polymerase chain reaction (PCR) products amplified with genomic DNA from trichostrongylid nematodes worms obtained in current study. Lane 1: PCR products of *Trichostrongylus colubriformis*; Lane 2: *T. vitrinus*; Lane 3: *Teladorsagia circumcincta*; Lane 4: Negative control; Lane 5: Positive control; and Lane M: 100-bp DNA marker

A 328 bp segment of the ITS2 gene was successfully amplified from each of the male worms (Fig. 3). The sequences were compared with different sequences available in GenBank database, by using the BLAST system.

**Fig. 4:**
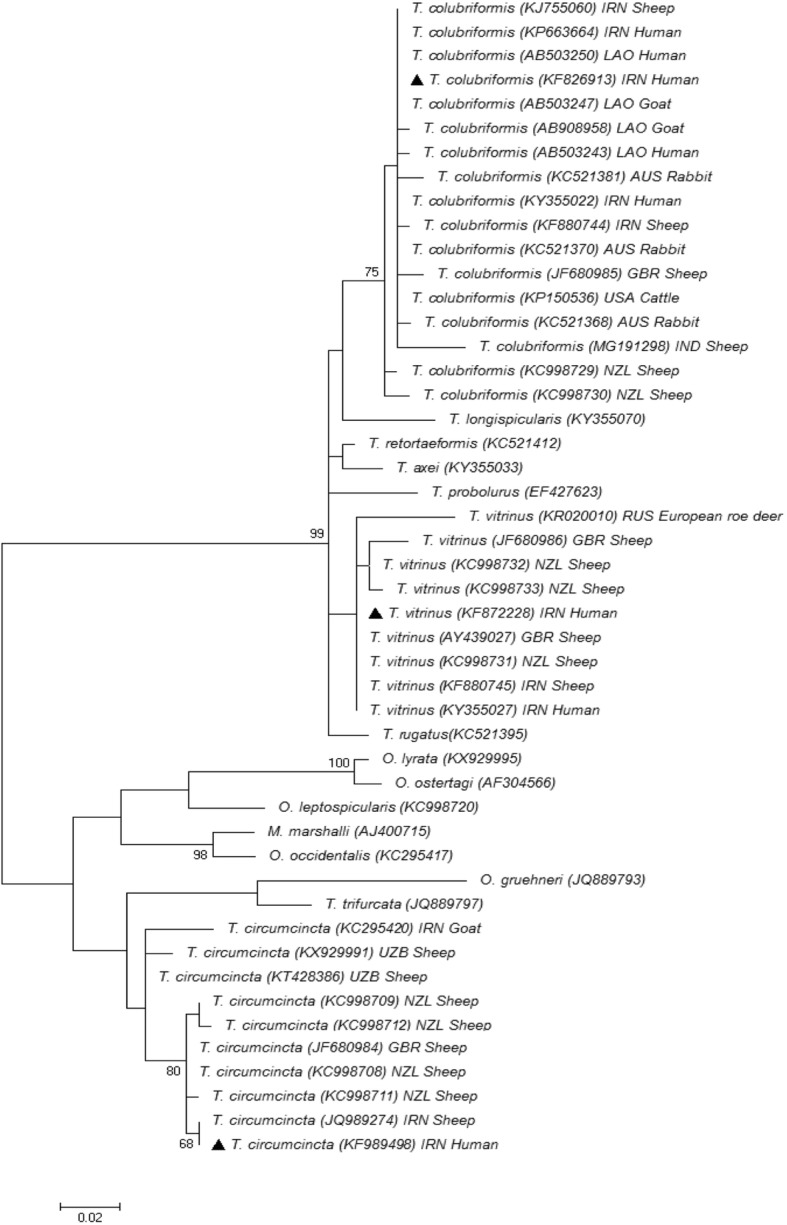
Phylogenetic tree of isolates of *Trichostrongylus* spp. and *Teladorsagia circumcincta* obtained in current study (▲) and reference sequences from previous studies based on ITS2 nucleotide sequences and constructed using Tamura-Nei model in MEGA software version 6. Iran (IRN), Laos (LAO), Russia (RUS), United States (USA), United Kingdom (GBR), Australia (AUS), India (IND), Uzbekistan (UZB) and New Zealand (NZL) are represented with country codes (ISO 3166-1 a-3 codes)

The molecular and phylogenetic analysis confirmed the morphologically distinguished worms. Phylogenetic tree indicates that each species obtained in current study is placed together with the same submitted to GenBank database (Fig. 4).

The MEGA 6 software was used to calculate mean intra-species distance. The mean intra-species distance rate within specimens of *T. colubriformis*, *T. vitrinus* and *T. circumcincta* obtained in current study and those available in GenBank amounted to 0.7, 1.1, 1.5%, respectively; meanwhile, mean inter-species sequence differences among subfamily Trichostrongylinae and also between subfamily Ostertaginae were significantly higher, being 2.6 and 6.5%, respectively.

## Discussion

Human infection with *Trichostrongylus* species is common in many countries throughout the world, in particular those located in the Middle East, Far East, and some African countries [5]. It has also a long history in Iran with the highest infection rates in the world that is most probably due to people’s habit of living in close contact with their livestock in rural regions and nomadic style of life in some parts of the country [2]. In addition, use of animal feces as fertilizer, consuming fresh wild grown aromatic plants, collecting from environment by villagers, as a part of human diet in many endemic areas and preparing parts of needed fuel from animal dung could be regarded among the other possible risk factors of human trichostrongylosis in endemic areas of Iran.

In recent years, the prevalence of most human geohelminths especially *Ascaris lumbricoides* and hookworms are sharply decreased over time in Iran [25, 26]; however, *Strongyloides stercoralis* [27–29] and *Trichostrongylus* spp. [6, 7, 14] are still reporting in some parts of the country. In 1961 prevalences of ascariasis in Kermanshah, Kurdistan, Isfahan, Guilan, Mazandaran, Khuzestan, West Azerbaijan and East Azerbayjan provinces were 69, 80, 80.6, 67.9, 86.3, 48.8 and 48.2% respectively. In all above mentioned areas the prevalence of ascariasis has decreased to less than 1% at present time. For example, In Mazandaran province, the prevalence of the disease in 1961, 1974, 1992, 2004, 2012 and 2016 have been reported as 86.3, 23, 1.2, 0.3, 0.4 and 0.6% respectively [25, 30, 31].

The same situation is seen in other parts of the country and a decreasing trend is also present for other geohelminthes. On the other hand, very rare cases of hookworm infection and ascariasis are reported in recent years while cases of strongyloidosis and trichostrongylosis are more encountered in some regions especially in northern provinces. The prevalences of 9.7% [32], 4.9% [33] and 0.9% [34] have been reported for strongyloidosis in Khuzestan, Mazandaran and Guilan provinces, respectively. In a different study carried out in Guilan province, 42% of hypereosinophilic individuals referred to the parasitology laboratory of Faculty of Medicin of Guilan University of Medical Sciences were found to be infected with *S. stercoralis* [35]. In addition, various studies verify the presence of human cases of trichstrongylosis in Iran in recent years. According to these studies the prevalences of human trichstrongylosis vary from 0.4 to 18% in some parts of the country [6, 9, 10, 30].

Infection with ten species of *Trichostrongylus* including *T. colubriformis* [2], *T. orientalis* [2], *T. vitrinus* [2], *T. skrjabini* [2], *T. axei* [2], *T. probolurus* [2], *T. lerouxi* [12], *T. capricola* [13], *T. longispicularis* [6], and an unnamed species [36] have been reported from humans in different parts of Iran. In the past decades, predominant species infecting humans were *T. orientalis* and *T. colubriformis* in most areas of the country such as central and southwestern parts. *T. orientalis* had low prevalence and intensity in herbivorous animals in these regions, which suggests that this species is predominantly a human parasite which is also prevalent in Far East, in particular in Japan, Korea and China [2, 37].

The using of human excreta, or night soil as fertilizer in agriculture in the past might be responsible for high prevalence of *T. orientalis* in these regions because the transmission of this parasite is mainly from human to human [2]. On the other hand, *T. colubriformis* was second most common species among inhabitants in the areas of Iran where it was also the most prevalent in domestic animals, and consequently can be considered to be mainly a zoonotic species [2]. Currently, it is not clearly known which species is prevalent among individuals in these regions.

The prevalence of *T. vitninus* in animals has been higher than that of *T. colubriformis* in most parts of the country, while the rates of trichostrongylosis due to *T. colubniformis* in humans have been reported to be higher than those due to *T. vitninus* [2]. It might be ascribed to higher susceptibility of humans to *T. colubriformis*. In north of Iran, *T. colubriformis* followed by *T. axei* and *T. vitrinus,* were reported most frequently in infected individuals in the past whereas the number of *T. orientalis* was usually low [2]. In these patients, the predominant species with high intensity was *T. colubriformis*. Gholami et al. (2015) and Sharifdini et al. (2017) have identified *T. colubriformis* as the most probable common species and *T. axei* in human in Mazandaran province, north of Iran using DNA detection in stool samples [15]. Recently, Sharifdini et al. (2017) reported *T. colubriformis*, as predominant species*, T. vitrinus*, *T. axei* and *T. longispicularis* in inhabitants of an endemic area in Guilan province. In the recent study, the authors reported the occurrence of natural human infection by *T. longispicularis* for the first time in the world [6]. Also, *T. colubriformis* was predominant among *Trichostrongylus* species infecting humans in Thailand [38], France [39] and Laos [4, 8]. This may be due to high prevalence of this species in herbivorous animals and also its high zoonotic potential [2]. Similar to the present study, human infections with *T. vitrinus* had been reported in the past from most parts of Iran, Armenia and Egypt [2]. Recently, *T. vitrinus* were detected in inhabitants of Fouman district in Guilan province [6].

Members of *Ostertagia* genus are found in the abomasum of cattle, sheep, goats and cervids worldwide. *O. ostertagi* is a cosmopolitan parasite of cattle. It is found with a lesser extent in sheep, goats, wild ruminants and horses [1, 40]. It was also reported occasionally in humans from Iran and Azerbaidjan [3]. *T. circumcincta* is the most important species of the genus found in sheep but it occurs in a variety of other ruminants such as goats and camels. This species has been reported from sheep, goats, wild sheep and camels in different parts of Iran [1, 18, 41–44]. Naem et al. [41] showed that 38% of sheep of Mazandaran province, located at the eastern part of Guilan, were infected with this parasite [41]. *T. circumcincta* has been reported only once to infect an inhabitant in Azerbaijan of former Soviet Union and hence this is the second report of human infection with this parasite [5].

Molecular phylogenetic analysis is a helpful tool to gain information on evolutionary relationships between the organisms. The existence of genetic variation among *Trichostrongylus* nematodes has been confirmed previously [6, 7, 20]. There are a few studies which have analyzed molecular-phylogenetic characterization of human trichostrongylosis [6, 7, 45, 46]. In this study, phylogenetic analysis represented that all *Trichostrongylus* species and *T. circumcincta* were clearly separated and there were only few differences among the species isolated from our study and those obtained from human and livestock subjects in other countries. Sequence of *T. colubriformis* specimen obtained in current study is placed in one branch together with isolates from Iran obtained from human (KP663664 and KY355022) and sheep (KJ755060), Laos (AB503247) goat isolate, Laos (AB503250) human isolate, Australia (KC521370) rabbit isolate and USA isolate from cattle (KP150536).

Furthermore, the phylogenetic analysis shows that *T. vitrinus* specimen of the current study has the same sequence with *T. vitrinus* obtained from human (KY355027) and sheep (KF880745) in Iran, and also sheep from Great Britain (AY439027) and New Zealand (KC998731). On the other hand, the phylogenetic tree represents that *T. circumcincta* specimens obtained in current study is placed together with sheep isolate from Iran (JQ989274). Although, the phylogenetic tree indicates that *T. colubriformis*, *T. vitrinus* and *T. circumcincta* are placed in separate branches, nevertheless, there is a high homology between sequences of human and livestock isolates. This result along with the other epidemiological and molecular studies reveals that zoonotic transmission is the most common route for these species.

## Conclusions

The finding of current study verifies the presence of *Trichostrongylus* species and *T. circumcincta* in inhabitants of Guilan province. Phylogenetic analysis shows that *Trichostrongylus* species and *T. circumcincta* isolated from human and animal have close associations and this verifies livestock as the main source of human infections. Consumption of fresh contaminated vegetables, close contact with livestock and their fecal materials and lack of personal hygiene in some rural regions might be the most recognized risk factors in the study area. Considering the current trend in growing use of animal dung as fertilizer in organic farms, the incidence of human infections with trichostrongyloid nematodes may increase in the future. In present study, the occurrence of natural human infection by *T. circumcincta* is reported for the first time in Iran and the second time in the world.

## Data Availability

The data supporting the conclusion of this article are included within the text and additional files. The sequences were deposited in GenBank database under the accession numbers KF826913, KF872228 and KF989498.

## Abbreviations

ITS2: Internal transcribed spacer 2
PCR: Polymerase Chain Reaction
rDNA: Ribosomal DNA
